# The Use of Sentinel Lymph Node Biopsy in* BRCA1/2* Mutation Carriers Undergoing Prophylactic Mastectomy: A Retrospective Consecutive Case-Series Study

**DOI:** 10.1155/2018/1426369

**Published:** 2018-01-01

**Authors:** Sara Câmara, Daniela Pereira, Saudade André, Beatriz Mira, Fátima Vaz, Rodrigo Oom, José Carlos Marques, João Leal de Faria, Catarina Rodrigues dos Santos

**Affiliations:** ^1^Department of Gynecology and Obstetrics, Hospital Dr. Nélio Mendonça, Avenida Luís de Camões, No. 57, 9004-514 Funchal, Portugal; ^2^Department of Pathology, Instituto Português de Oncologia Francisco Gentil (IPOLFG), Lisbon, Portugal; ^3^Department of Breast Cancer Risk Evaluation Clinic and Department of Medical Oncology, Instituto Português de Oncologia Francisco Gentil (IPOLFG), Lisbon, Portugal; ^4^Department of Surgical Oncology, Instituto Português de Oncologia Francisco Gentil (IPOLFG), Lisbon, Portugal; ^5^Radiology Department, Instituto Português de Oncologia Francisco Gentil (IPOLFG), Lisbon, Portugal

## Abstract

**Introduction:**

Sentinel lymph node biopsy in prophylactic mastectomy is controversial. It avoids lymphadenectomy in occult carcinoma but is associated with increased morbidity. Women with BRCA mutations have a higher incidence of occult carcinoma and our objective was to assess the clinical utility of sentinel lymph node biopsy when these women undergo prophylactic mastectomy.

**Materials and Methods:**

Seven-year retrospective consecutive case-series study of women, with a BRCA deleterious mutation, admitted to prophylactic mastectomy, at our center. Breast MRI < 6 months before surgery was routine, unless contraindicated.

**Results:**

Fifty-seven patients (43% BRCA1; 57% BRCA2) underwent 80 prophylactic mastectomies. 72% of patients had had breast cancer treated before prophylactic mastectomy or synchronously to it. The occult carcinoma incidence was 5%, and half of the cases were invasive. SLNB was performed in 19% of the prophylactic mastectomies; none of these had tumor invasion. Women with invasive carcinoma who had not undergone sentinel lymph node biopsy were followed closely with axillary ultrasound. The median follow-up was 37 months, with no local recurrence; 1 patient died of primary tumor systemic relapse.

**Conclusions:**

Our data do not support this procedure for routine (in agreement with previous literature), in this high risk for occult carcinoma population.

## 1. Introduction

Prophylactic mastectomies have become more frequent [1, 2], and there are several possible reasons for this. These include new predictive models, the identification of gene mutations other than* BRCA* (e.g.,* PALB2*,* CHEK2*, and others), the increased diagnosis of high-risk lesions, and the so-called “Angelina Jolie effect” [3–6].

In women with a* BRCA1/2* deleterious mutation, contralateral prophylactic mastectomy is expected to improve all-cause mortality [7, 8]. Meanwhile, there is no evidence of a survival benefit with bilateral prophylactic mastectomies (either in* BRCA* mutation carriers or other high-risk women) [9]. There is also no evidence of any benefit of proceeding to sentinel lymph node biopsy in prophylactic mastectomy, nor any evidence about how to proceed when occult carcinoma is found during prophylactic mastectomy and axillary staging is no longer possible.

Sentinel lymph node biopsy is considered to have minimal surgical risk and avoids axillary lymphadenectomy if an occult carcinoma is found [10, 11]. However, it is time-consuming and expensive and may cause complications including paresthesias, lymphedema, and arm motion limitation (with estimated risks of 9%, 2–7%, and 4%, resp.) [12–15]. With a low probability of occult carcinoma in prophylactic breast surgery [16] and a low probability of lymph node metastasis [17], sentinel lymph node biopsy has been estimated to benefit only 2.8% of patients [18]. A recent meta-analysis has recommended against the routine use of sentinel lymph node biopsy [16]. A positive sentinel lymph node biopsy in the absence of occult carcinoma has also been described [17, 19–25], and crossover metastasis was a possible explanation [16, 17, 21–24].

Considering the higher incidence of prophylactic mastectomies and the present controversy on sentinel lymph node biopsy in this type of surgery, our study adds to the literature by specifically addressing this subject in a high risk for occult carcinoma women,* BRCA1/2* mutation carriers, and to whom preoperative breast magnetic resonance imaging (MRI) was routine. Our objective was then to review the outcomes and clinical utility of sentinel lymph node biopsy in prophylactic mastectomy in a consecutive group of systematically evaluated high risk for occult carcinoma women.

## 2. Materials and Methods

We performed a retrospective analysis of* BRCA1*/2 mutation carriers who underwent at least 1 prophylactic mastectomy at our center, between 2009 and 2015. All the patients had previously been counseled at our center's Breast Cancer Risk Evaluation Clinic.

The study population descriptive analysis included age, genetic diagnosis, breast cancer history (no personal history of breast cancer, breast cancer treated in the past or a synchronous to prophylactic mastectomy, histology, clinical stage, and previous oncological treatments), surgery performed, breast and axillary histological findings, and follow-up.

All patients underwent breast MRI within 6 months before prophylactic mastectomy, unless contraindicated. Breast MRI was carefully reviewed by our Breast Cancer Committee Radiologists, in order to exclude infraclinical or contralateral carcinoma. Any prophylactic side doubtful lesion would undergo biopsy or SLNB would be directly indicated (according to the level of suspicion and the risks of postulating surgery).

Prophylactic mastectomies were total or skin-sparing (with or without nipple sparing) mastectomies, with or without immediate reconstruction. A dual technique (technetium 99 sulfur colloid and patent blue dye) was used for sentinel lymph node biopsy.

Both prophylactic mastectomy type of surgery and the indication for SLNB were indicated by the Breast Cancer Committee (including dedicated radiologists, medical oncologists, and surgeons). The choice of prophylactic mastectomy type is beyond the scope of this work. The indication for SLNB took into consideration the patient informed consent after discussing the benefits and risks of alternative procedures and the risk factors for occult carcinoma.

For pathological examination, the breast parenchyma was entirely processed in 3-4 mm slices and the sentinel lymph node was microscopically evaluated in 2 mm slices using hematoxylin-eosin staining (according to our prophylactic mastectomy and sentinel lymph node biopsy protocol). During this research, all specimens were reviewed by 2 pathologists to confirm the histological findings.

To determine the clinical utility of sentinel lymph node biopsy, the occult carcinoma and the sentinel lymph node biopsy positivity rates were calculated. Patient and tumor characteristics were analyzed to identify positive predictive factors for occult carcinoma or sentinel lymph node biopsy positivity. For follow-up outcomes, local recurrence and mortality were analyzed. Our study protocol was multidisciplinary defined and accepted by our Institution Committee.

## 3. Results

**Figure 1:**
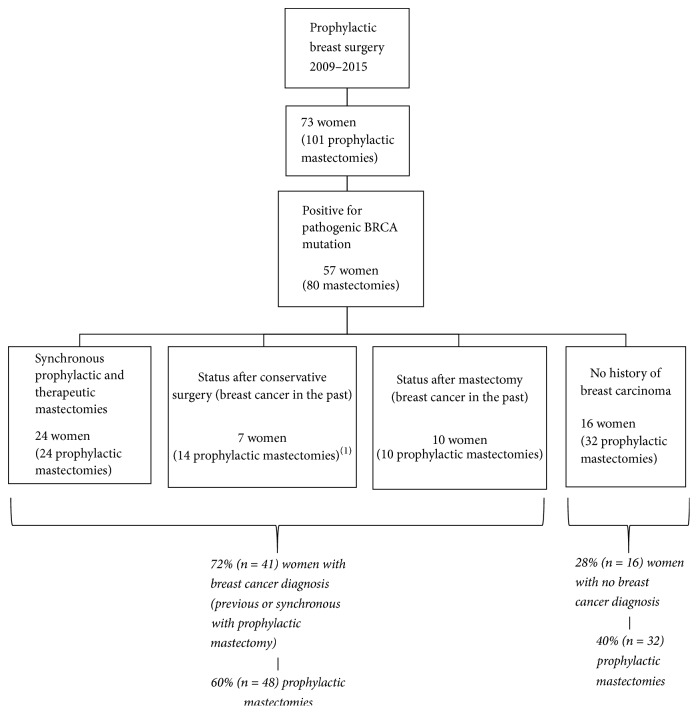
*Patients admitted to prophylactic breast surgery* ((1) including prophylactic mastectomy of the remnant breast).

From a total of 73 women (101 prophylactic mastectomies), 57 patients (80 prophylactic mastectomies) were confirmed to be* BRCA1*/*2* deleterious mutation carriers and were considered for further analysis (Figure 1).

MRI was performed within 6 months before prophylactic mastectomy in 82% (*n* = 47) of the patients. The remaining patients either refused the examination, were allergic to the MRI contrast agent, or had other MRI contraindications (Table 1).

Half of the women (51%) had been treated with chemotherapy, radiotherapy, or hormonotherapy before undergoing prophylactic mastectomy. These therapies were either neoadjuvant treatment for breast cancer that was being operated on simultaneously with the prophylactic mastectomy or treatment for a previous breast cancer. Women who had previously undergone surgery for breast cancer had a median time interval between breast cancer surgery and prophylactic mastectomy of 90 months (interquartile range [IQR] 10–228 months). No locally advanced or metastatic breast cancer was present at the time of prophylactic mastectomy. Two patients underwent surgery for recurrent breast cancer at the same time as their contralateral prophylactic mastectomy (Supplementary Table 1).

The occult carcinoma rate was 5% (*n* = 4), and the occult invasive carcinoma rate was 2.5% (*n* = 2). Occult carcinoma occurred in 75% of patients with a personal history of breast cancer and a* BRCA1* pathogenic mutation, although this was not a significant result because of the small number of cases. Among the 4 patients with occult carcinoma, this was present at a small volume of disease and there were no relevant similitudes in the characteristics of breast cancer (if there was a previous or synchronous breast cancer diagnosed before surgery), radiological findings on the prophylactic side, or occult carcinoma histological findings. During a median follow-up of 30 months (IQR 25–55 months), no local or distant recurrence was diagnosed (Table 2).

A sentinel lymph node biopsy was performed in 19% (*n* = 15) of the prophylactic mastectomies. Median number of SLN removed per operated breast was 1 (1-2).

The sentinel lymph node was identified in all of the cases, and none was positive for tumor cells. Nonsentinel lymph nodes were not retrieved in any of the cases. In our center, the rate of sentinel lymph node biopsy in prophylactic mastectomy was at its highest in 2015 (71% of the cases). Two women with occult invasive carcinoma had not undergone sentinel lymph node biopsy during prophylactic mastectomy and were managed with both axillary ultrasound and close follow-up.

With no patients (*n* = 57) lost to follow-up, at a median of 37 months (14–57), 1 death had occurred because of a primary tumor in the contralateral breast (a 45-mm,* not otherwise specified*, poorly differentiated, triple-negative, invasive carcinoma) and systemic relapse. There were no breast or axillary recurrence events (both prophylactic and therapeutic sides).

## 4. Discussion

Sentinel lymph node biopsy is controversial in the prophylactic mastectomy setting because of the expected advantage of avoiding axillary lymphadenectomy versus the low number of cases it will benefit [26]. Studies addressing this question have included a heterogeneous population of high-risk women. In addition to including only* BRCA1/2* mutation carriers, other notable characteristics of our study included the facts that an MRI evaluation before surgery is part of our standard protocol and that whole specimens of the breast parenchyma were entirely processed and the slides reviewed to confirm histologic findings. Our study population is the result of a systematized assessment by both a Breast Cancer Risk Evaluation Clinic and a Breast Cancer Committee.

First of all, in what regards MRI, others have argued against its routine use in prophylactic mastectomy, because of its high cost and low sensitivity for the detection of occult carcinoma [22]. However, MRI is recommended for the surveillance of* BRCA1/2* mutation carriers [27, 28]; MRI has a high negative predictive value for invasive carcinoma [29] and can be useful for selecting women who do not need to undergo sentinel lymph node biopsy (because occult carcinomas that have not been identified using MRI have a lower probability of lymph node metastasis) [30]. Our group reinforces the importance of MRI at a short interval before surgery, since all our patients had a* BRCA1/2 *pathogenic mutation and, therefore, a higher risk both for occult and for interval carcinoma. In fact, in spite of being a high-risk group, the occult carcinoma incidence in our study (5%) was similar to that reported by others (6.0% [3], 4.8% [10], and 1.76% [31]) and the same can be concluded for our occult invasive carcinoma rate (2.5% versus 2% [17], 1.2% [10, 31], and 0% [3]).

In what concerns the sentinel lymph node biopsy rate (19%), it was lower than other reported rates (21.8% [31], 29% [22], 66.7% [3], 74% [30], and 76.9% [17]). However, for the follow-up described in our study, the patient outcome was not affected by the decision to undergo sentinel lymph node biopsy and the verified death was because of primary tumor progression, with no local recurrence events.

For the evaluation of possible positive predictive factors, this could not be accomplished, considering we found no positive sentinel lymph nodes. In the literature, sentinel lymph node biopsy positivity was associated with contralateral breast cancer, especially if the disease was locally advanced or inflammatory, and with a higher number of positive lymph nodes [3, 16–18, 21–23, 25, 32]. Sentinel lymph node biopsy positivity was also associated with lymphovascular or skin/nipple involvement, higher histologic grades [21], patients aged over 60 years, and lobular histology [26]. Of relevance, in our group of patients there was no locally advanced breast cancer case at the time of prophylactic mastectomy.

The decision to do not proceeding to lymphadenectomy in patients with occult invasive cancer without sentinel lymph node biopsy has been described by others [19, 31] and, in our perspective, close follow-up (comprising an axillary ultrasound) is an option which avoids overtreatment or lymphadenectomy-associated morbidity.

The median number of lymph nodes removed from operated breasts was 1 (range 1-2), which is in agreement with the results of other studies (1.35 [10], 1.46 [25], and 2 [31]). This assessment is important because a higher number of biopsied lymph nodes reduce the false negative rate but also increase morbidity.

Limitations of our study include its retrospective nature, the short follow-up period, and the fact that we did not analyze complications related to sentinel lymph node biopsy (due to an underreport perception). Unfortunately, a high level of evidence on this subject is hard to achieve.

Finally, regarding our findings and the results in the literature, we recommend that sentinel lymph node biopsy should be offered to a* selected* group of patients, including women with a synchronous or previous breast cancer diagnosis and those who cannot be evaluated using MRI in the short interval before surgery.

## 5. Conclusion

Our data and present evidence support that performing a sentinel lymph node biopsy in selected patients, instead of routine sentinel lymph node biopsy, is the better approach for prophylactic mastectomy, including in women with a deleterious mutation in* BRCA1/2.* Even if recent routine preoperative breast MRI is probably the most important strategy in the selection of these patients, other factors must be considered.

## Figures and Tables

**Table 1:** Descriptive analysis of the prophylactic mastectomy population.

Patient characteristics	Number of patients (*N* = 57)	%
*Age (years), median (IQR)*	43.8 (37–51)	
*BRCA mutation*		
(i) *BRCA1*	25	43
(ii) *BRCA2*	32	57
*MRI evaluation prior to surgery *	47	82

Surgery description	Number of mastectomies (*N* = 80)	%

*Breast: total mastectomy*		100
(i) Type of prophylactic surgery		
Total mastectomy	4	5
Skin (+/− nipple) sparing mastectomy	76	95
(ii) Type of reconstruction		
*Latissimus dorsi* flap and definitive breast implant	54	68
Subpectoral breast implant or tissue expander	25	31
Transverse rectus abdominis muscle flap	1	1
*Axilla*		
(i) Sentinel lymph node biopsy	15	19%

IQR: interquartile range.

**Table 2:** Description of occult malignancy cases (*N* = 4).

	Case number 1	Case number 2	Case number 3	Case number 4
Age (on the day of prophylactic mastectomy)	34 years	41 years	40 years	32 years

Genetic risk	*BRCA1*	*BRCA1*	*BRCA2*	*BRCA1*

Surgery indication	Prophylactic + therapeutic	Prophylactic + therapeutic	Bilateral prophylactic	Contralateral prophylactic

Breast cancer characteristics *(therapeutic side or breast cancer in the past)*	(i) Invasive, not otherwise specified, histologic grade 2 (ii) Estrogen receptor 100%. Progesterone receptor 10% Cerb2 negative (iii) cT1N0 (iv) pT1cN0(sn)	(i) Invasive, not otherwise specified carcinoma, histologic grade 2 (ii) Triple negative (iii) cT1N0 (iv) pT2N0(sn)	-	*2 years before prophylactic mastectomy*(i) Invasive not otherwise specified carcinoma, histologic grade 3 (ii) Triple negative (iii) cT3N1 (iv) Neoadjuvant chemotherapy. Radical mastectomy. Radiotherapy *(unknown pathological response to chemotherapy)*

Mammography/ultrasound	No findings	No findings	Dense pattern	Stable asymmetry

MRI (prophylactic side)	BI-RADS 2	BI-RADS 2	BI-RADS 2 (bilaterally)	*Contraindication for MRI*

Time interval from MRI to prophylactic mastectomy	103 days	84 days	97 days	*-*

Occult carcinoma histological findings	Ductal carcinoma in situ (6 mm)	Ductal carcinoma in situ (10 mm)	Lobular microinvasive and lobular in situ multifocal Estrogen receptor in in situ component-positive	Invasive, not otherwise specified (2 mm) Estrogen and progesterone receptor-negative

Sentinel lymph node biopsy	Not done	Not done	Not done	Not done

Follow-up	(i) 24 months (ii) No local or distant recurrence	(i) 63 months (ii) No local or distant recurrence	(i) 30 months (ii) No local or distant recurrence	(i) 29 months (ii) No local or distant recurrence

BI-RADS: Breast Imaging-Reporting and Data System Classification.

## Supplementary Materials

Supplementary Materials Supplementary Table  1. Tumor characteristics and oncologic treatments received—other than surgical—in women with breast cancer (either synchronous to prophylactic mastectomy or prior to it).
