# Supplementary material for: The Use of Sentinel Lymph Node Biopsy in BRCA1/2 Mutation Carriers Undergoing Prophylactic Mastectomy: A Retrospective Consecutive Case-Series Study
Source: Int J Breast Cancer. 2018 Jan 1;2018:1426369. doi: 10.1155/2018/1426369 (PMC5817815; doi:10.1155/2018/1426369)
Supplement: Supplementary Materials — Supplementary Table 1. Tumor characteristics and oncologic treatments received—other than surgical—in women with breast cancer (either synchronous to prophylactic mastectomy or prior to it). [file 1426369.f1.pdf]

Tumor characteristics	**Synchronous prophylactic and therapeutic mastectomies** N=24 (100%)	**Breast cancer in the past (post mastectomy or post conservative)** N=17 (100%)
**Histology**		
- Ductal *in situ*	6 (25)	1 (6)
- Invasive (*not otherwise specified*)	18 (75)	16 (94)
**Clinical Stage**		
- 0	5 (20)	1 (6)
- I	10 (42)	3 (18)
- II	7 (29)	1 (6)
- III	0 (0)	5 (29)
- IV	0 (0)	0 (0)
- Unknown	2 (14)	7 (41)
**Other treatments**	*(only neoadjuvant)*	*(neo – or adjuvant)*
- Chemotherapy	4 (17)	10 (59)
- Radiotherapy	0	10 (59)
- Hormonotherapy	0	8 (47)
- No data	-	5 (29)
**Time interval between surgeries**	-	90 months ± 56 (10–228)

Supplementary Table 1. Tumor characteristics and oncologic treatments received - other than surgical – in women with breast cancer (either synchronous to prophylactic mastectomy or prior to it)
